# Composite inorganic membranes containing nanoparticles of hydrated zirconium dioxide for electrodialytic separation

**DOI:** 10.1186/1556-276X-9-271

**Published:** 2014-05-29

**Authors:** Yuliya S Dzyazko, Yurii M Volfkovich, Valentin E Sosenkin, Nadejda F Nikolskaya, Yurii P Gomza

**Affiliations:** 1 VI Vernadskii Institute of General & Inorganic Chemistry, Palladin Pr. 32/34, Kiev 03142, Ukraine; 2 AN Frumkin Institute of Physical Chemistry & Electrochemistry, Leninskii Pr. 31, GSP-1, Moscow 119071, Russia; 3 Institute of Macromolecular Chemistry of the NAS of Ukraine, Kharkivske sh., 48, Kiev 02000, Ukraine

**Keywords:** Composite ion exchange membranes, Hydrated zirconium dioxide, Nanoparticles, Standard contact porosimetry, Electrodialysis

## Abstract

The aim of the work was to elucidate the nature of charge-selective properties of macroporous composite inorganic membranes modified with nanoparticles of hydrated zirconium dioxide. The membranes have been investigated using methods of standard contact porosimetry, potentiometry, electron microscopy and small-angle X-ray scattering. The ion exchanger has been found to deposit inside pores of ceramics. Differential curves of pore volume distribution have been resolved using Lorentz functions; each maximum has been related to structure elements of the matrix and ion exchanger by means of calculations according to homogeneous and heterogeneous geometrical models. It was found that the voids, the radius of which is 4 to 8 nm, are responsible for charge selectivity of the composite membranes. These pores are formed due to blocking of macropores of ceramics with aggregates of nanoparticles of the ion exchanger; the radius of these aggregates is 20 to 24 nm. The membranes were applied to desalination of the solution containing NaCl. The removal degree of the salt from the solution reached 95% and 9% for the composite and unmodified membranes, respectively.

## Background

Inorganic membranes can operate at high temperatures and in aggressive media; moreover, they are stable against fouling with organic matters [1,2]. Since these materials possess remarkable properties, they are attractive for separation processes particularly for electromembrane techniques [3]. However, application of ceramic separators to electromembrane processes is limited by an absence of charge selectivity in spite of a nanoporous active layer. This is due to extremely low ion exchange capacity (low surface charge density) of ceramics, since these materials are produced at high temperature [4], which does not provide retention of functional groups.

Earlier, we modified Al_2_O_3_-ZrO_2_ ceramics with hydrated zirconium dioxide (HZD), which contains -OH groups. HZD is able to sorb cations (Cat) in alkaline media [5]

(1) $$-O^{-}H^{+}+{\mathrm{Cat}}^{+}↔-O^{-}{\mathrm{Cat}}^{+}+H^{+}$$

and anions (An) in acidic solutions:

(2) $$-\mathrm{OH}+\mathrm{HAn}↔-{{\mathrm{OH}}_{2}}^{+}{\mathrm{An}}^{-}.$$

The conditions of thermal treatment of the membranes provided ion exchange ability of HZD.

Pores of 190 nm dominated in pristine ceramics. After modification, their size decreased to 80 nm [6,7] indicating formation of an active layer inside the pores of ceramics, opposite to known inorganic materials for baromembrane separation [1,2]. This location of the active layer provides its mechanical durability.

Predominant pores of the composite membranes [6,7] cannot provide overlapping of intraporous diffusion double electric layers. In spite of this, the membranes were shown to possess charge selectivity. They demonstrate membrane potential in rather concentrated acid solutions [6]. When the composite separators are applied to electrodialysis, the ion transport through these separators is due to migration of counter ions and electrolyte diffusion during electrodialysis [7]. At the same time, no migration of co-ions through these separators was found.

Many types of ceramics contain larger pores (up to several microns) in comparison with the material investigated in [6,7]. The aims of the work involve formation of the inner active layer in coarse-pored membranes, ascertainment of the cause of their charge selectivity and application of these materials to electromembrane separation.

A method of standard contact porosimetry (SCP) was applied to membrane investigation. The method allows us to obtain pore size distribution in a wide interval of 1 nm to 300 μm as well as total volume of micropores of 0.3 to 1 nm [8-11]. The SCP method is non-destructive, since it does not require high pressure compared to mercury porosimetry. Thus, small pores can be determined more exactly. Moreover, analysis of integral pore size distribution gives a possibility to determine particle size using geometrical models [12-14]. However, in the case of composites, the particle size of their components can be close to each other; as a result, the constituents cannot be recognized. Thus, the next important task of the work is to develop an approach for analysis of pore size distributions for composite materials.

## Experimental

### Synthesis of the composite membranes

Planar ceramic membranes (matrix) based on TiO_2_ (TAMI GmbH, Hermsdorf, Germany), which contain no active layer, were used. Sol of insoluble zirconium hydroxocomplexes was prepared by adding a NH_4_OH solution (1,000 mol m^−3^) to a 1 M ZrOCl_2_ solution (1,000 mol m^−3^) at 353 K followed by boiling for 48 h and storage for 48 days at 298 K [6,7]. Sol was analysed with a dynamic light-scattering method using a Zetasizer Nano ZS device (Malvern Instruments, Worcestershire, UK). Stability of particle distribution has been found after long-term storage.

The membrane was impregnated with sol, treated with a NH_4_OH solution (1,000 mol m^−3^), dried at ≈ 298 K and heated at 423 K [6,7]. A layer of the ion exchanger was removed from the outer surface of the membrane with ultrasonic activation at 30 kHz. The procedure, which involves impregnation, HZD deposition, drying, heating and ultrasonic treatment, was repeated two and seven times. The samples were marked as TiO_2_ (matrix), TiO_2_-HZD-2 and TiO_2_-HZD-7 (modified membranes). Similar growth of HZD content (2.2 to 2.4 mass%) was reached both for TiO_2_-HZD-2 (in comparison with the matrix) and TiO_2_-HZD-7 (in comparison with TiO_2_-HZD-2).

### Electron microscopy

After dehydration of sol at room temperature, its solid constituent was investigated using a JEOL JEM 1230 transmission electron microscope (JEOL Ltd., Tokyo, Japan). Finely dispersed powders obtained both from initial and modified membranes were also researched. Before the investigations, the powders of ceramics were treated with a CH_3_COOH solution (100 mol m^−3^) to shade the modifier particles.

Transverse section of the membranes was investigated using a Zeiss EVO 50XVP scanning electron microscope (Carl Zeiss AG, Oberkochen, Germany).

### Small-angle X-ray scattering

Finely dispersed powders of the membranes were inserted into cuvettes, the thickness of which was 0.1 to 0.2 mm, with 17-μm-thick Mylar windows. Small-angle X-ray scattering (SAXS) curves were obtained in a vacuum Kratky camera using a Cu-anode tube. Recording of SAXS data has been carried out under the conditions of multiple scanning of a scintillation detector at scattering angles of 0.03° to 4.0°. The first treatment of the SAXS data was carried out by means of the FFSAXS11 program. The exclusion of parasitic scattering by the camera and cuvette windows, normalization of the scattered intensity to absolute units, and the introduction of the collimation correction were performed.

### Standard contact porosimetry

The membranes were heated at 423 K before the measurements. Octane was used as a working liquid [8-11]. The curves of differential pore volume (*V*) distribution ($\frac{\mathrm{dV}}{d\left(\log r\right)}$, where *r* is the pore radius) were resolved by Lorentz components using the PeakFit v. 4.12 program. Treatment of the curves involved resolution within the intervals of pore radius of 1 to 100 nm and 1 to 10^5^ nm and comparison of the data for peaks with a maximum at ≈ 100 nm. Data adequacy is confirmed by coincidence of these maxima in two diapasons and high correlation coefficient (0.99). This procedure was necessary because the $\frac{\mathrm{dV}}{d\left(\log r\right)}$ values are rather low at 1 to 100 nm.

The particle density of the membranes (*ρ*_p_) was determined using a pycnometer method [15], and the bulk density (*ρ*_b_) was estimated from geometrical parameters.

### Sorption capacity and potentiometric measurements

Ion exchange capacity of the membranes has been determined by their treatment with a HCl solution (100 mol m^−3^), washing with deionized water followed by treatment with a NaOH solution (100 mol m^−3^) and analysis of the eluate using an I-160 M potentiometer and Cl^−^-selective electrode. The solution was neutralised with HNO_3_ before the measurements.

Membrane potential (*E*_m_) was measured at 298 K using a two-compartment cell [16,17]. HCl solutions (10 and 15 to 100 mol m^−3^) filled their chambers, where Ag/AgCl electrodes were placed. Transport numbers of counter ions (*t*_m_) through the membrane were calculated as [16]

(3) $$E_{m}=-\frac{\mathrm{RT}}{F}\left[\ln\frac{a_{1}}{a_{2}}\pm 2\int_{a_{1}}^{a_{2}}\left(1-t_{m}\right)d\ln a_{\pm }\right],$$

where *a*_1_ and *a*_2_ are the activities of counter ions in less and more concentrated solutions, respectively; indexes ‘+’ and ‘−’ correspond to cations and anions, respectively; *R* is the gas constant; *F* is the Faraday constant; *T* is the temperature; and *a*_±_ is the activity of ions in a solution of varied concentration*.* The equation is valid for a 1:1 electrolyte like HCl. The transport numbers of counter ions (Cl^−^) were found from a derivative of the function, which describes a deviation of the membrane potential from theoretical value $\left(\frac{\mathrm{RT}}{F}\ln\frac{a_{1}}{a_{2}}\right)$:

(4) $$2\frac{\mathrm{RT}}{F}\left(1-t_{Cl^{-}}\right)=\frac{d\left(E_{м}-\frac{\mathrm{RT}}{F}\ln\frac{a_{1}}{a_{2}}\right)}{d\ln a_{\pm }}.$$

The difference of $E_{м}-\frac{\mathrm{RT}}{F}\ln\frac{a_{1}}{a_{2}}$ was found, and then its dependence on *a*_±_ (i.e. on activity of more concentrated solution, *a*_2_) was plotted. At last, the transport number was calculated from a slope of the curve.

### Electrodialysis

**Figure 1:**
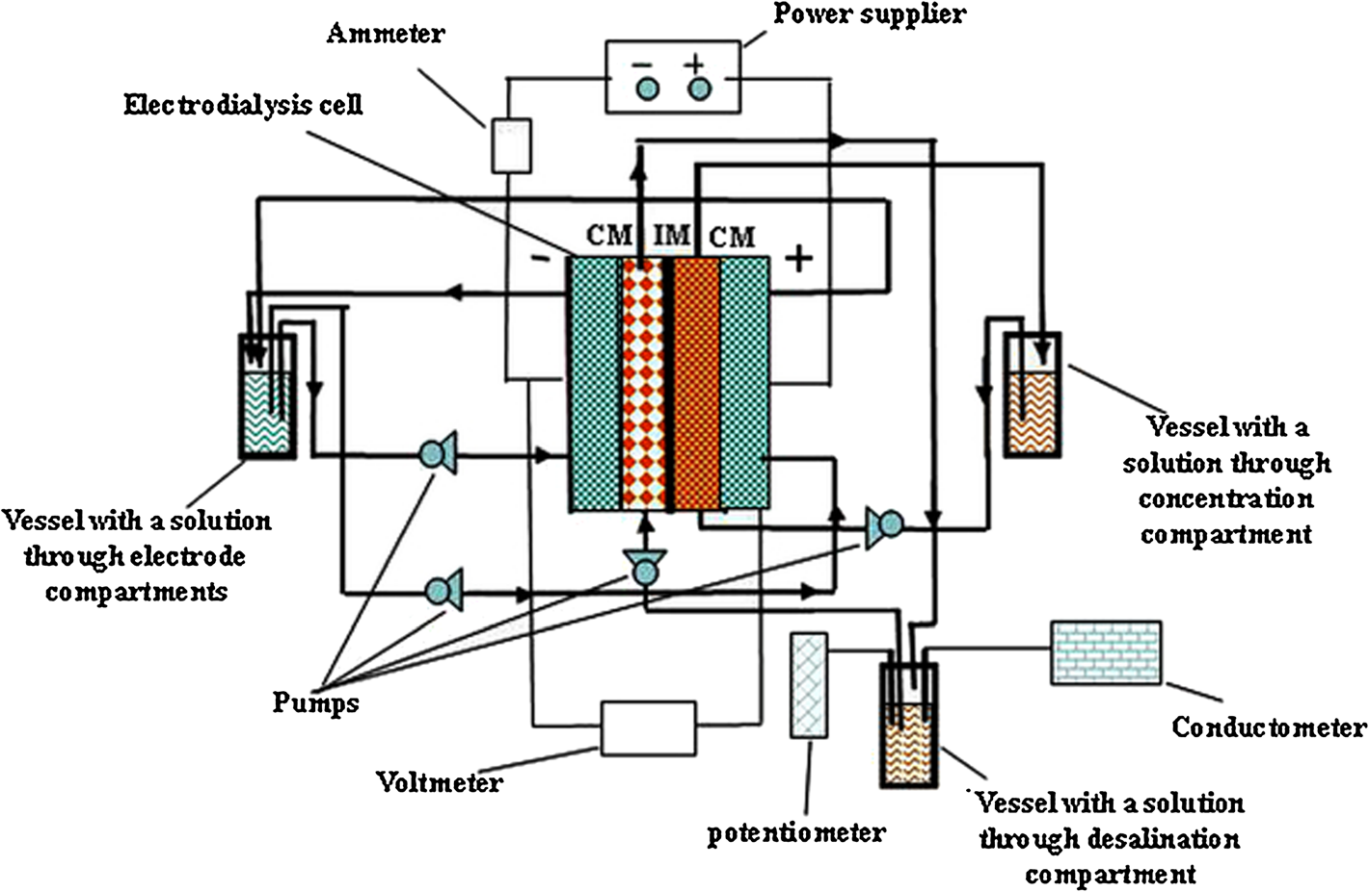
Scheme of the electrodialysis setup.

The experimental setup involved a four-compartment cell, three independent liquid lines, power supplier and measurement instrumentation described earlier [7] (Figure 1). A scheme of the membrane system was as follows: cathode compartment, polymer cation-exchange membrane (Nafion 117, Dupont, Wilmington, DE, USA), desalination compartment filled with glass spacers (6 × 10^−4^ m of a diameter), inorganic membrane, concentration compartment, polymer cation-exchange membrane and anode compartment. The distance from each membrane to the other (and from cation-exchange membrane to the opposite electrode) was 1 cm, the cross-sectional area of each compartment was 4 cm, and the effective area of each membrane was 16 cm (4 cm × 4 cm).

A solution containing NaCl (10 mol m^−3^), the volume of which was 50 cm^3^, circulated from the desalination compartment with a flow velocity of 1 cm^3^ s^−1^ (first liquid line). The second line provided circulation of the solution, which contained initially K_2_SO_4_ (1,000 mol m^−3^), through the cathode and anode compartments (second line). At last, a H_2_SO_4_ solution (100 mol m^−3^) circulated through the concentration compartment. The content of Cl^−^ and Na^+^ species in the solution being purified was controlled by means of ion-selective electrodes. The removal degree of NaCl from the solution was calculated as $\frac{C_{i}-C}{C_{i}}\times 100\%$, where *C* is the concentration at time *τ* and *C*_i_ is the initial concentration. The current efficiency was calculated as $\frac{\mathrm{zFn}}{\mathrm{iA\tau}}\times 100\%,$ where *z* is the charge number, *n* is the amount of electrolyte removed from the solution, *i* is the current density and *A* is the membrane area.

## Discussion

### Sol of zirconium hydroxocomplexes

**Figure 2:**
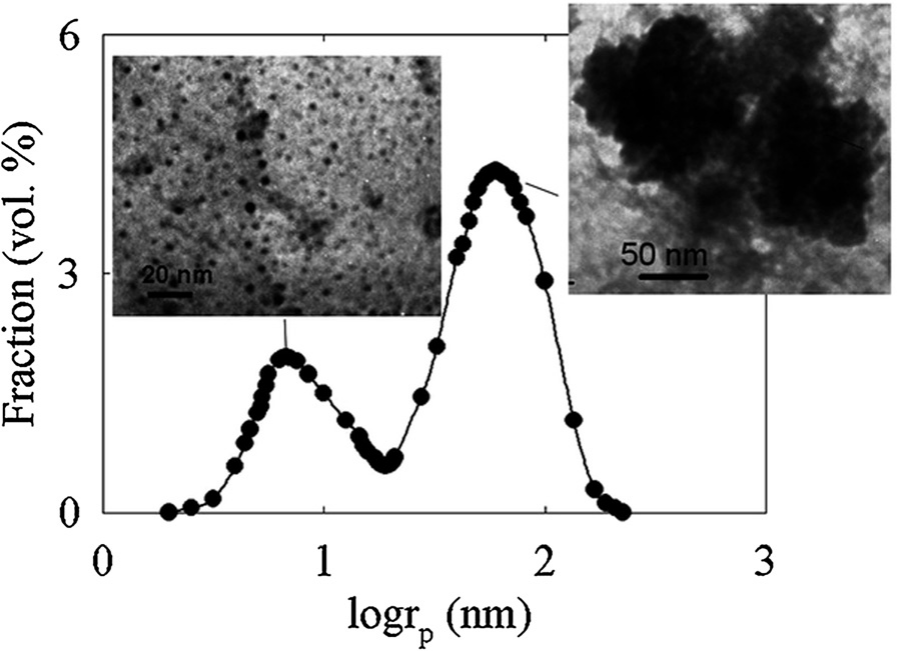
**Particle size distribution in sol of insoluble zirconium hydroxocomplexes.** Insets: TEM images of the solid constituent of dehydrated sol. Left corner, single nanoparticles; right corner, aggregated nanoparticles.

Figure 2 illustrates distribution of particle size in sol. The curve demonstrates two maxima at *r*_*p*_ = 7.5 nm (particles I) and 60 nm (particles II). Minimal particle radius has been found as 2 nm. Different particles of the solid constituent of sol are seen in the inset of Figure 2. The smallest nanoparticles are ideally spherical. The shape of particles II is also close to spherical, but their surface is rough.

During sol formation, fragmentation and defragmentation of nanoparticles occur simultaneously [18]. As a result, sol can contain several types of particles [19]. The first one is non-aggregated particles; their merging leads to formation of larger ones.

### Structure of membranes

**Figure 3:**
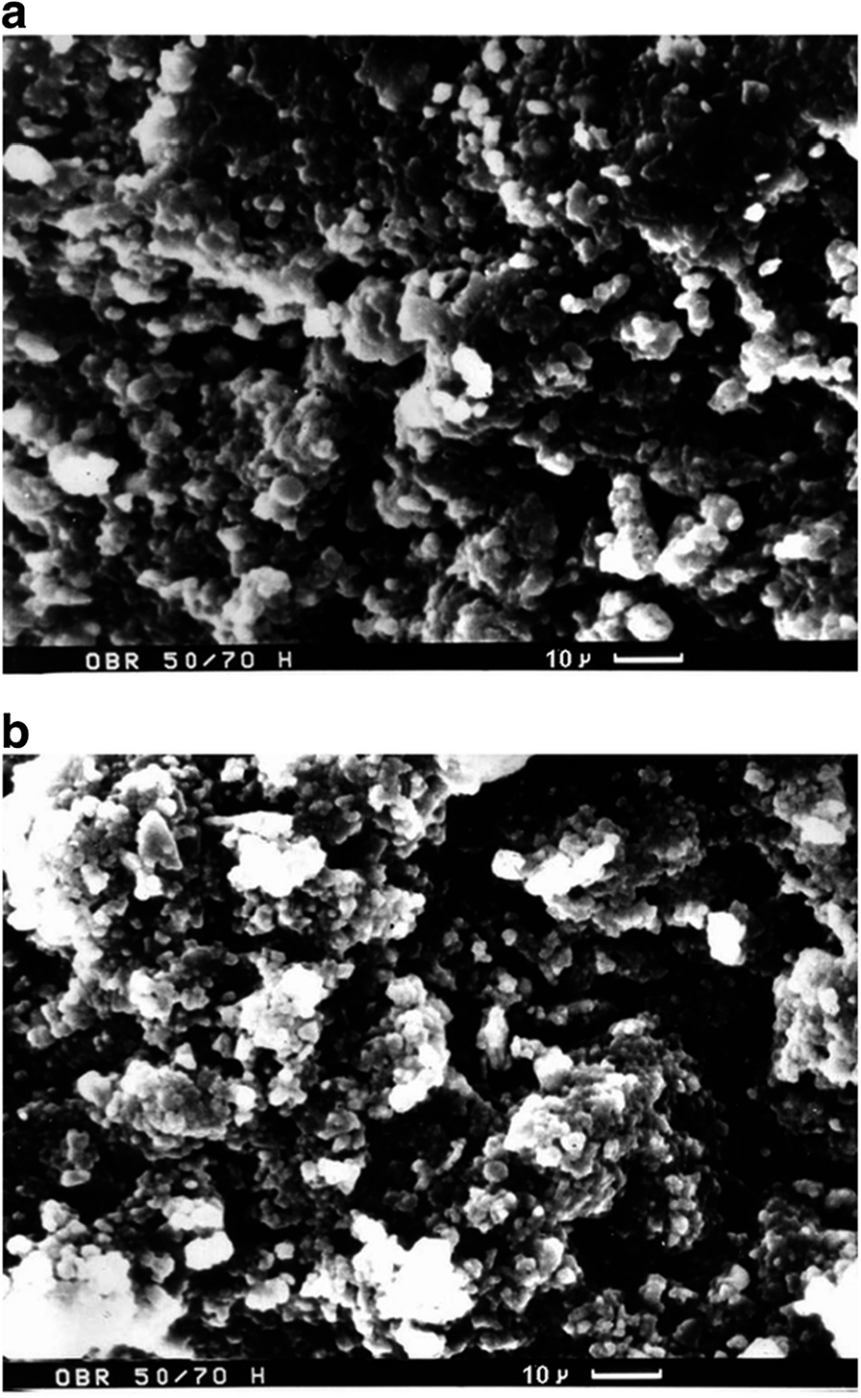
**SEM image of transverse section of initial (a) and modified (b) membranes.** Particles of ceramics, the shape of which is close to spherical, are visible **(a)**, and aggregates of HZD particles are seen inside pores of the matrix **(b)**.

**Figure 4:**
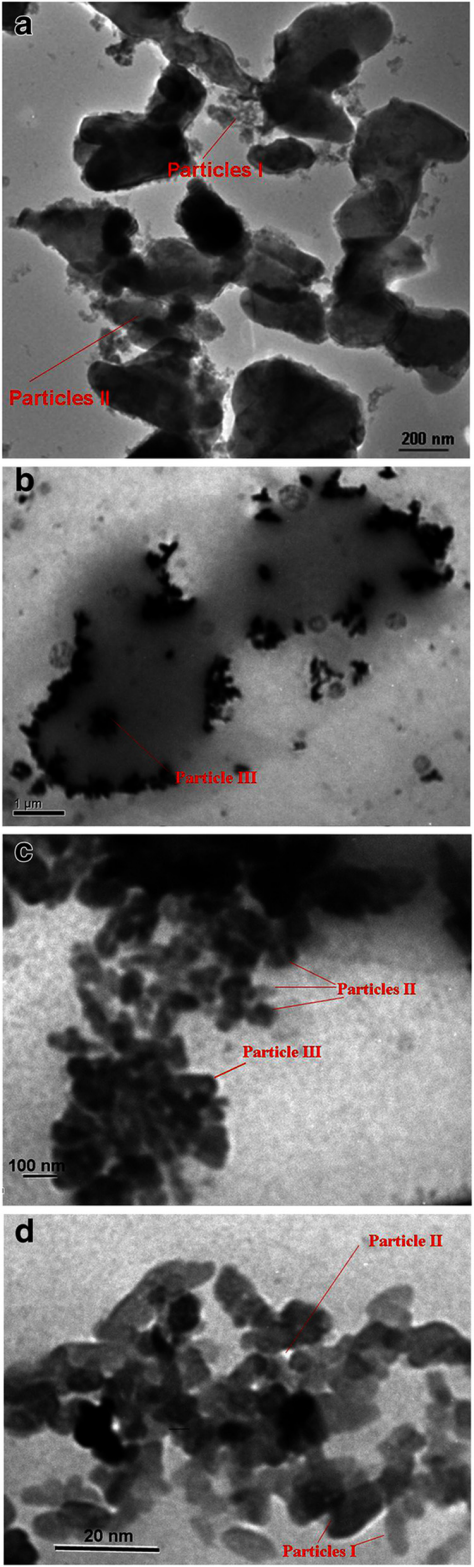
**TEM images of powder of pristine (a) and modified membranes (b-d).** Particles I and II of ceramics are visible **(a)**. HZD particles, which are shaded with CH3COOH, are seen on the surface of particles of ceramics **(b-d)**: particles III (b), II and III (c), and I and II (d) are visible.

Spheres of micron size are seen in the scanning electron microscopy (SEM) image of the TiO_2_ sample (Figure 3a). The particles are distorted due to annealing and pressure during ceramics preparation. Widening and narrowing of spaces between the globules are also visible. Globular HZD particles on the internal surface of the membrane are seen for the TiO_2_*-*HZD-2 sample (Figure 3b). However, increase of the matrix mass after modification is inconsiderable (Table 1).The transmission electron microscopy (TEM) image of powder of the pristine membrane is given in Figure 4a. No smaller constituents are visible inside the particles. We can separate three types of particles of the ceramics. The first type includes nanosized particles (particles I); the particles, the radius of which is about 100 nm, are related to the second type (particles II). The third type is the particles of micron size (particles III). Aggregates of particles I and II are located on the surface of particles III.Figure 4b,c,d shows TEM images of powder of the modified membrane. The aggregates of HZD particles (several hundreds nanometers, particles III), which were shaded by organic acid, are visible on the surface of micron particles of ceramics (grey clouds), as seen in Figure 4b. These aggregates include smaller ones, the size of which is about 100 nm (particles II) (Figure 4c,d). At last, these aggregates consist of nanoparticles (particles I). Their shape is close to spherical but distorted, opposite to the sol constituent due to thermal treatment of the composite membrane.

**Table 1:** Porous structure of the composite membranes

**Sample**	**Increase of mass,%**	***S***_**m**_**, m**^**2 **^**kg**^**−1**^		**Density, kg m**^**−3**^		**Volume of micropores, cm**^**3 **^**g**^**−1**^	***ϵ***_m_**,** $\epsilon_{m}^{/}$
		**Total**	**Micropores**	*ρ*_p_	*ρ*_b_		
TiO_2_		820	80	4,260	3,270	3.0 × 10^−4^	0.23
TiO_2_-HZD-2	2.4	3,340	990	4,260	3,350	1.5 × 10^−3^	0.21
TiO_2_-HZD-7	4.6	10,430	5,120	4,260	3,420	5.0 × 10^−3^	0.20

**Figure 5:**
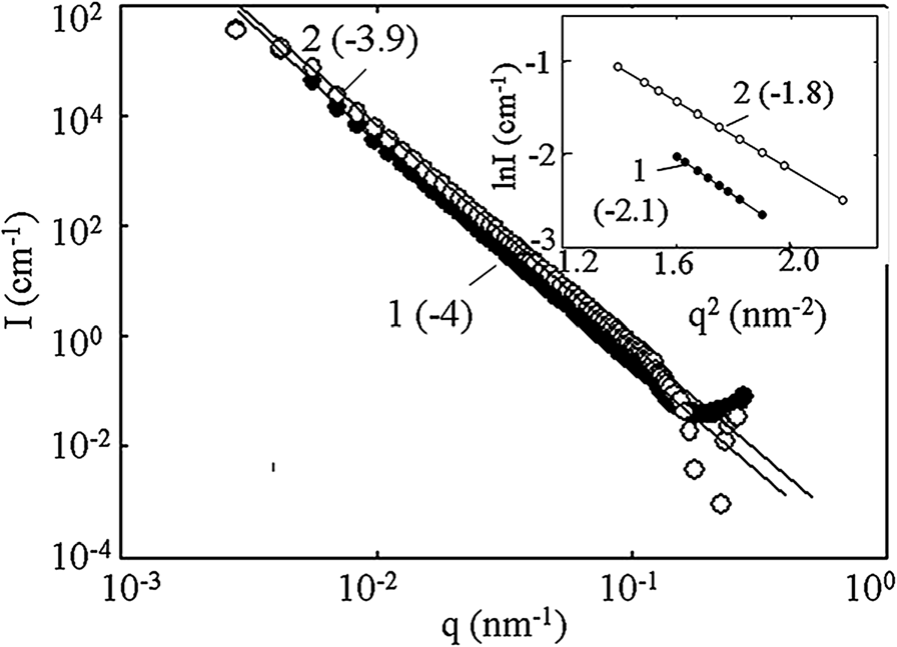
**Intensity as a function of scattering vector.** Inset: logarithm of intensity as a function of *q*^2^. Materials: pristine (1) and modified (2) membranes. Slopes of the linear parts of the curves are given in brackets.

The SAXS data (Figure 5) allow us to determine the average particle sizes. The size of the smallest particles I of the ceramic matrix can be estimated according to the Guinier formula [20]:

(5) $$\ln I=2\ln\left(\Delta ,\rho \right)-\frac{R_{g}^{2}q^{2}}{3}$$

where Δ*ρ* is the difference of electron densities between the particle and its environment, and *R*_g_ is the gyration radius, which has been determined from the slope of the linear part of ln*I* − *q*^2^ curve at *q* = 1.1 to 1.6 nm^−1^ (inset of Figure 5). The particle radius (*r*_p_) was calculated as 1.29*R*_g_[21,22]. It was found, that *r*_p_ *=* 3 nm.

The log*I −* log*q* curve (where *I* is the intensity, *q* is the scattering vector), which has been obtained for pristine ceramics, is characterized by a long straight part within the interval of scattering vector of 2.82 × 10^−2^ to 1.1 nm^−1^. This interval corresponds to particles II of the ceramic matrix. The slope of the curve is −4; this indicates smooth surface of these particles, which include no constituents [21,22]. The curves demonstrate deviation from linearity under low *q* values; thus, the order of particle size is about 100 nm. Larger particles cannot be determined with a SAXS method.

Regarding the modified membranes, a small change of the slope of the linear part (*q =* 2.82 × 10^−2^ to 1.1 nm^−1^) has been found. Thus, deposition of the modifier on particles II is inconsiderable. However, a change of slope of the ln*I* − *q*^*2*^ curve at wider angles indicates the presence of HZD particles, which are smaller, than particles I of the matrix.

### Porosity measurements

The results obtained with a pycnometer method allow us to determine porosity of the samples. Modification of the matrix causes an increase of bulk density of the membranes; however, no change of particle density has been found. Thus, the particle densities of the ion exchanger and matrix are equal. Porosity (*ϵ*_m_ for the initial matrix and $\epsilon_{m}^{/}$ for the modified membranes) has been calculated as $1-\frac{\rho_{p}}{\rho_{b}}$[15]. The porosity decreases in the order: TiO_2_ > TiO_2_-HZD-7 > TiO_2_-HZD-2*.*

**Figure 6:**
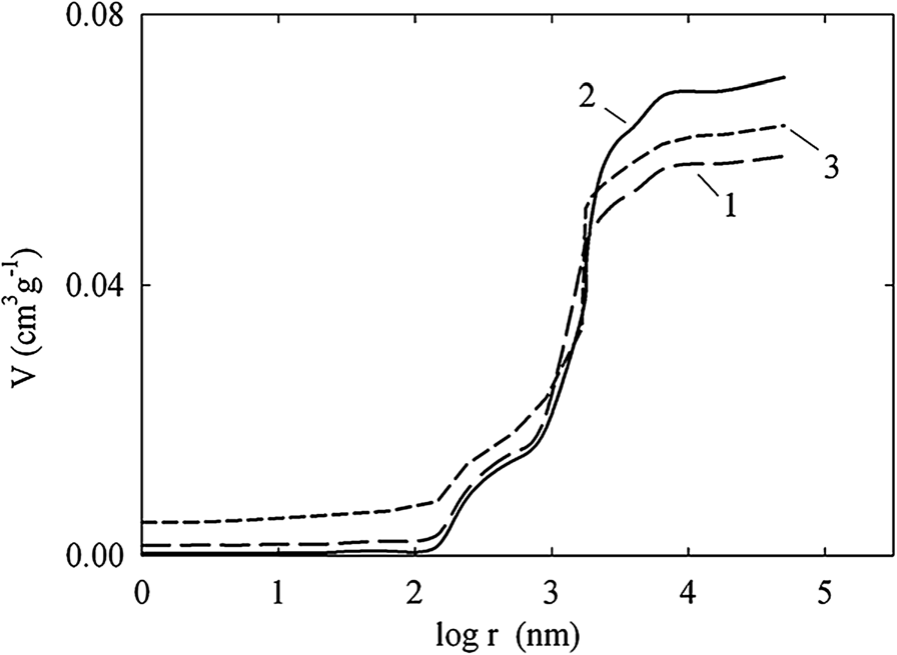
**Integral distribution of pore volume for TiO**_**2 **_**(1), TiO**_**2**_**-HZD-2 (2) and TiO**_**2**_**-HZD-7 (3) samples.**

Integral pore distributions, which have been obtained with the SCP method, are plotted in Figure 6. The curves demonstrate low (*r =* 1 to 100 nm) and rapid increase of pore volume (*r* > 100 nm), indicating preferable macroporous structure of the membranes. However, micropores, which can be found as the curve intersection with ordinate axis, are also visible. Micropores provide 10% (TiO_2_), 30% (TiO_2_-HZD-2) and 55% (TiO_2_-HZD-7) of the total membrane surface (*S*_m_) (see Table 1).

The ratio of $V_{\mathrm{micr}}^{/}-V_{\mathrm{micr}}$ values is 1:3.9 for TiO_2_-HZD-2 and TiO_2_-HZD-7 membranes, respectively (here, *V*_micr_ and $V_{\mathrm{micr}}^{/}$ are the volume of micropores for pristine and modified membranes, respectively). The ratio of $\frac{m^{/}-m}{m}$ (here, *m* and *m*^*l*^ are the mass of matrix and modified membrane, respectively) is 1:1.9. This is evidently due to different porous structures of HZD: more compact structure is attributed to the TiO_2_-HZD-2 sample.

The volume of the ion exchanger in mass unit of the membrane has been estimated as $\frac{\left(\epsilon_{m}^{/}-\epsilon_{m}\right)m^{/}}{\rho_{b}}$, and the porosity of the HZD layer $\left(\bar{\epsilon }\right)$ was calculated using the expression:

(6) $$\bar{\epsilon }=\frac{\left(V_{\mathrm{micr}}^{/}-V_{\mathrm{micr}}\right)\rho_{b}}{\mathrm{m\pi}\left(\epsilon_{m}^{/}-\epsilon_{m}\right)}.$$

More compact HZD structure has been also found for the TiO_2_-HZD-2 membrane (Table 2). The surface of the ion exchanger was assumed to be proportional to the mass growth of membranes.

**Table 2:** Parameters of globular model for the matrix and ion exchanger layer

**Parameter**	**Homogeneous model**			**Heterogeneous model**			
	**Matrix**	**Ion-exchanger**		**Spheres**	**Matrix**	**Ion-exchanger**	
		**TiO**_**2**_**-HZD-2**	**TiO**_**2**_**-HZD-7**			**TiO**_**2**_**-HZD-2**	**TiO**_**2**_**-HZD-7**
*ϵ*, $\bar{\epsilon }$	0.23	0.29	0.46		-	-	-
*S*, m^2^ kg^−1^	820	1.05 × 10^5^	2.09 × 10^5^	-	-	-	-
*ϵ*_p_	-	-	-	I	-	0.03	0.42
				II	0.02	0.26	0.04
				III	0.21		
Packing	CFC or HXG	CBC	SC	I	-	CBC	SC
				II	CFC or HXG		
				III		-	-
$$S\frac{\epsilon }{\epsilon_{p}}$$ , $S\frac{\bar{\epsilon }}{\epsilon_{p}}$, m^2^ kg^−1^	-	-	-	I		7.77 × 10^5^	2.27 × 10^5^
				II	8,176	3.06 × 10^4^	3.88 × 10^4^
				III	201	-	-
*r*_*g*_, nm	859	7	4	I	-	5	3
				II	86	23	20
				III	3,500	-	(≈400)
*r*_*n*_^a^, nm	133 (204)	1 (≤1)	1 (≤1)	I	-	1 (≤1)	1 (≤1)
				II	13 (8)	5 (8)	8 (4)
				III	542 (204)	-	(190)
*r*_*c*_^a^, nm	355 (1,730)	2 (2)	2 (2)	I	-	2 (2)	2 (2)
				II	36 (39)	9 (8)	13 (6)
				III	1,449 (1730)	-	(331)

^a^Experimental values identified according to pore size distributions are given in brackets.

**Figure 7:**
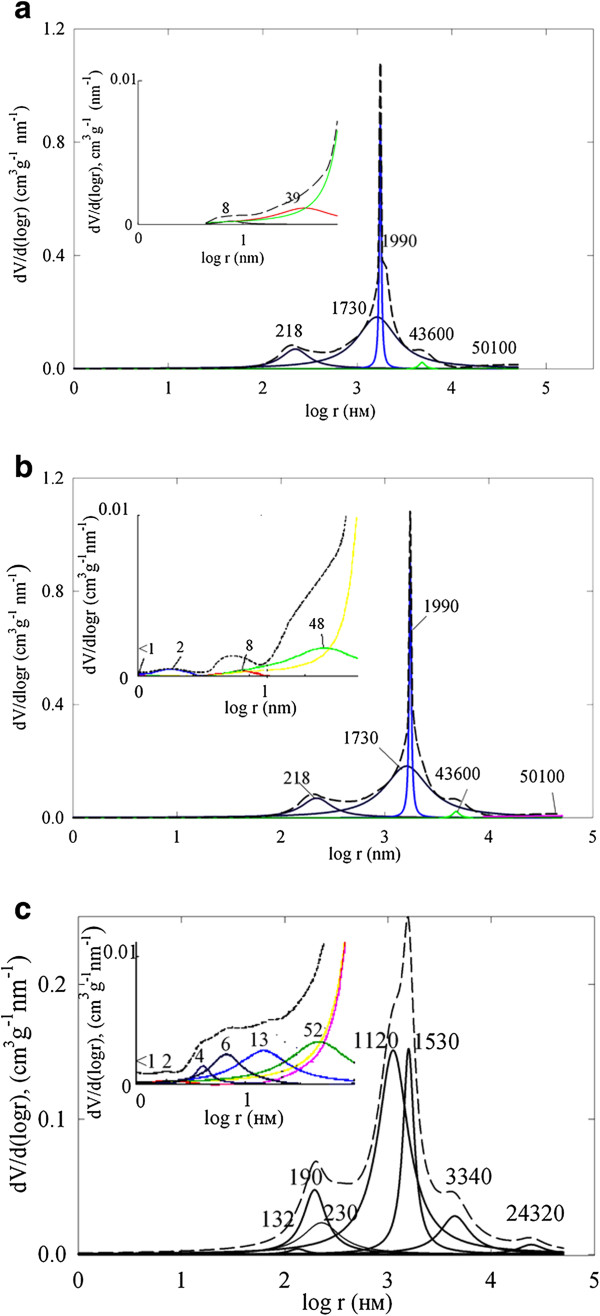
**Differential distribution of pore volume for TiO**_**2 **_**(a), TiO**_**2**_**-HZD-2 (b) and TiO**_**2**_**-HZD-7 (c) membranes.** Insets: enlarged distributions. Dashed curves correspond to experimental data, and solid curves are related to calculated peaks. Numbers are related to the site of maxima of the peaks (nm).

Differential distributions of pore volume are given in Figure 7. The *r* values are represented as log*r*; the peaks are symmetric. Thus, the plots can be resolved by Lorentz functions. Since $\frac{\mathrm{dV}}{d\left(\log r\right)}=2.3r\frac{\mathrm{dV}}{\mathrm{dr}}$, the peak area gives the pore volume caused by each type of particles.

### Calculation of porous structure according to globular models

Both homogeneous and heterogeneous globular models were applied to relate the maxima either to the matrix or to ion exchanger. The models have been developed by A.P. Karnaukhov; their main principles are described in [12-14]. Parameters of the models are radii of globules (*r*_p_), pore necks (*r*_n_) and pore cavities (*r*_c_); the values of surface and porosity are also used. The magnitudes of *r*_n_ and *r*_c_ are calculated using special factors for each type of globule packing: *r*_n_ = 0.41*r*_p_ and *r*_c_ = 0.73*r*_p_ for simple cubic (SC), *r*_n_ = 0.22*r*_p_ and *r*_c_ = 0.29*r*_p_ for body-centred cubic (BCC), and *r*_n_ = 0.15*r*_p_ and *r*_c_ = 0.41*r*_p_ for hexagonal (HXG) or face-centred cubic packing (FCC). A packing type is determined from the porosity (see Table 2).

According to the homogeneous model, the effective particle size was calculated as $r_{p}=\frac{3}{\rho_{p}S}$. The heterogeneous model provides analysis of integral pore size distributions [12-14]. Porosity caused by different types of particles is determined according to each semi-wave. In the case of composite materials, it is difficult to recognize their components, when sizes of the particles are close to each other. We have proposed resolution of differential pore size distributions by Lorentz components; these functions provide the best agreement of experimental and calculated curves. The globular model was assumed to give pairs of peaks: the first maximum corresponds to narrowing of pores between globules (pore necks), and the second one is related to their widening (pore cavities). Then, the porosity, which is attributed to the peak, was found by means of peak integration. The surface of each type of pores was found as $S\frac{\epsilon }{\epsilon_{p}}$ (matrix) and $S\frac{\bar{\epsilon }}{\epsilon_{p}}$ (ion exchanger), where *ϵ* or $\bar{\epsilon }$ are the total porosity, and *ϵ*_*p*_ is the porosity due to each type of particles.

Regarding the matrix, analysis of integral pore distributions allows us to recognize the smallest particles I; however, their size cannot be determined exactly. Particles III form pores, which give two maxima about 1,730 nm (pore cavities) and 218 nm (pore necks) (see Figure 7a). Two maxima at 39 and 8 nm correspond to pores caused by particles II. Three stripes at 1,990, 4,360 and 50,100 nm are outside the model since their areas becomes smaller with an increase of pore radius. These pores are evidently caused by irregular particles, which are seen in the SEM image (see Figure 3a). Experimental $\frac{r_{c}}{r_{n}}$ relation for particles III is larger than the calculated value probably due to compaction of the particles due to pressure and annealing; this can lead to deviation from the globular model. No influence of pressure and annealing has been found for smaller particles II: they are in an agreement with the model.Since both heterogeneous and homogeneous models show that the matrix structure is formed by particles III, the aggregates of particles II are evidently located on the surface of larger spheres. This assumption is confirmed by the TEM image of the matrix powder (see Figure 4a).

Two additional peaks (1 to 3 nm) due to HZD are visible for modified membranes (see Figure 7b,c). Calculations give nanosized particles I, which evidently form a structure of the ion exchanger (particles I). Similar results were obtained using the homogeneous model. These particles are evidently associated into aggregates (particles II); pores between them give maxima at 8 nm for TiO_2_-HZD-2 and 4 and 6 nm for TiO_2_-HZD-7. Evidently, there are only HZD aggregates inside the matrix, since the SAXS data indicate no considerable change of surface of particles II of the matrix. Indeed, the size of particles II of the modifier is larger than the pores, which are formed by particles II of the matrix. In the case of TiO_2_-HZD-2, the maxima for necks and cavities are overlapped with a peak attributed to the matrix and cannot be separated. A shift of the peak at 39 nm (TiO_2_) to 52 nm (TiO_2_-HZD-7) has been found. This indicates formation of larger particles III; their size can be estimated approximately from the peak at 52 nm, which is related to pore necks. These particles are evidently located in the cavities of pores, which are caused by the largest particles III of the matrix. The peaks at *r >* 100 nm for modified membranes are shifted towards lower *r* values in comparison with the matrix. This indicates HZD deposition inside macropores of the ceramics.

### Potentiometric transport numbers of counter ions

Potentiometric measurements give additional information about the membrane structure. No membrane potential (*E*_m_) has been registered for the matrix. *E*_m_ > 0 V in the case of modified samples. Since the membranes show anion exchange ability in acidic media [6,7], Cl^−^ and H^+^ species are considered as counter- and co-ions, respectively.

**Figure 8:**
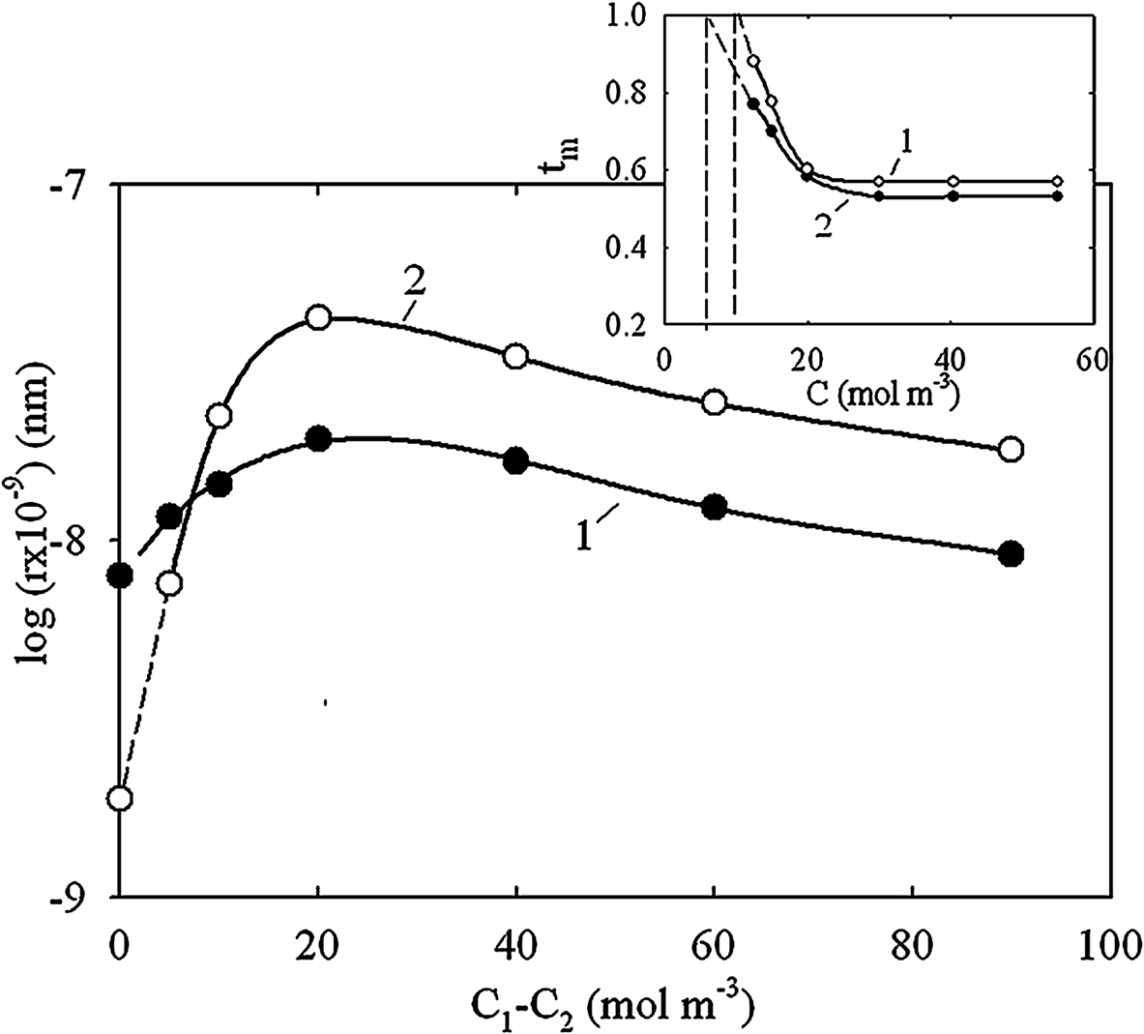
**Radius of pores, which determine charge selectivity, as a function of *C***_**1**_**-*C***_**2 **_**(calculations according to formula (**7**))**. Extrapolation of curves to the ordinate axis gives true value of the radius. Inset: transport number of counter ions as a function of average concentration of the solutions. Extrapolation of the curves to *t*_m_ = 1 gives the concentration at which the diffusion parts of intraporous double electric layers are overlapped. Membranes: TiO_2_-HZD-2 (1) and TiO_2_-HZD-7 (2).

The transport numbers of counter ions are higher than 0.5 (Figure 8). The following formula was applied to find the size of pores, which are responsible for charge selectivity [23]:

(7) $$t_{m}=t\left(1+\frac{\mathrm{FrC}}{\mathrm{k\eta}}\right){\left(t+\frac{\mathrm{FrC}}{\mathrm{k\eta}}\right)}^{-1},$$

where *t* is the transport number of Cl^−^ in a solution, *k* is the shape coefficient (*k =* 2.8 for pores between globules), *η* is the surface charge density and *C* is the average value of concentrations of the solutions from two sides of the membranes. The surface charge density was estimated from sorption measurements as 0.07 C m^−2^ (TiO_2_-HZD-2) and 0.18 C m^−2^ (TiO_2_-HZD-7).

Formula (7) gives the transport number at which concentrations of the solutions from two sides of the membrane (*C*_1_ and *C*_2_) are close to each other. The *r* value was plotted as a function of *C*_2_-*C*_1_. Extrapolation of the curve to *C*_2_-*C*_1_ *=* 0 evidently gives the ‘real’ *r* magnitude, which has been estimated as 8 (TiO_2_-HZD-2) and 2 (TiO_2_-HZD-7) nm (Figure 8).

It was also assumed that the transport number of counter ions can reach 1, if intraporous diffusion double electrical layers are overlapped. In this case, the radius of pores, which determine charge selectivity, can be calculated as [24]:

(8) $$r=\sqrt{\frac{\epsilon_{d}\epsilon_{0}\mathrm{RT}}{2F^{2}C}},$$

where *ϵ*_0_ is the dielectric permittivity of vacuum, and *ϵ* is the dielectric constant (80 for water). The concentration, which corresponds to *t*_m_ *=* 1, was found by extrapolation of *t*_m_ − *C* curves (inset of Figure 8). The *r* values were estimated as 7 nm (TiO_2_-HZD-2) and 4 nm (TiO_2_-HZD-7).

**Figure 9:**
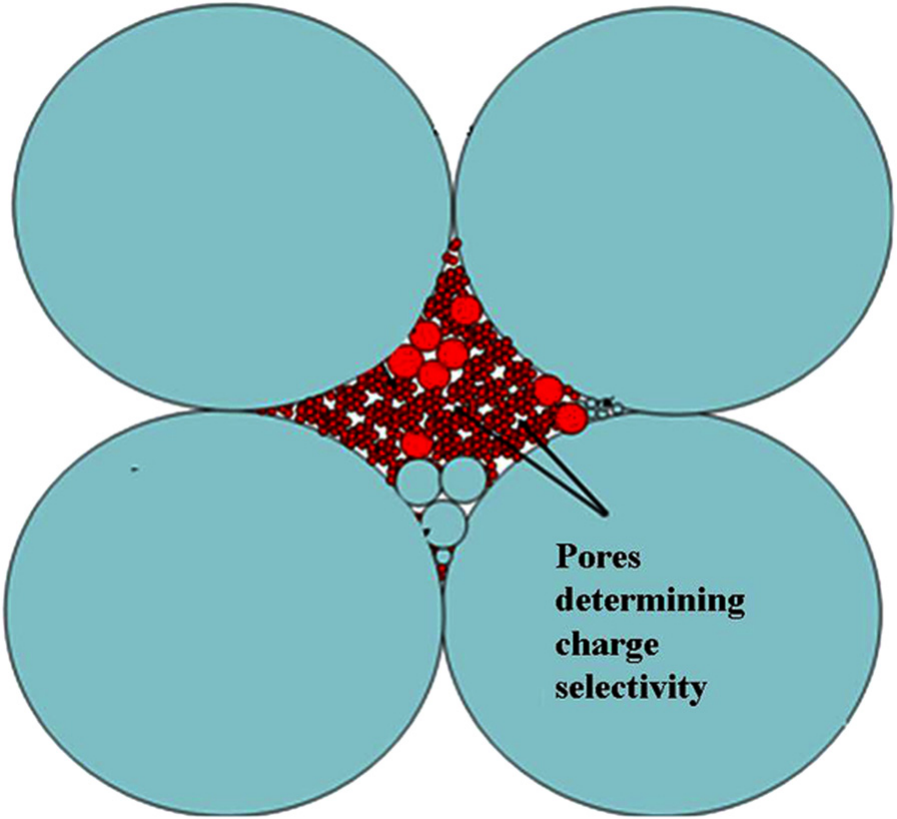
**Structure of composite membrane.** Blue circles = matrix; red-orange circles = ion exchanger. Pores between aggregates of particles of the ion exchanger are responsible for charge selectivity.

Analysis of the $\frac{\mathrm{dV}}{d\left(\log r\right)}-\log r$ curves shows that the Equations 7 and 8 give pore radius, which corresponds to peaks with maxima at 8 nm (TiO_2_-HZD-2) or 4 nm (TiO_2_-HZD-7). These peaks are attributed to necks of pores caused by particles II of the modifier, which evidently block pores of the matrix. Since intraporous diffusion double electrical layers are not overlapped at high concentration of the solution, the transport numbers of counter ions cannot reach 1. The transport number of counter ions is higher than 0.5 due to their excess in the diffusion part of the double electric layer [23].Based on data of electron microscopy, SAXS, porosimetry and potentiometric measurements, the structure of the composite membranes has been proposed. The matrix is formed by large particles of micron size; aggregates of smaller particles are placed on their surface (Figure 9). Matrix pores are blocked with aggregates of HZD nanoparticles.

These ‘corks’ isolate macropores, which are recognized with the porosimetry method as predominant. Large particles of sol can penetrate the matrix during the first modification procedure. After blocking of the matrix pores, only the smallest particles are able to enter the membrane; moreover, they form the loosening structure of the ion exchanger.

### Electrodialysis

**Figure 10:**
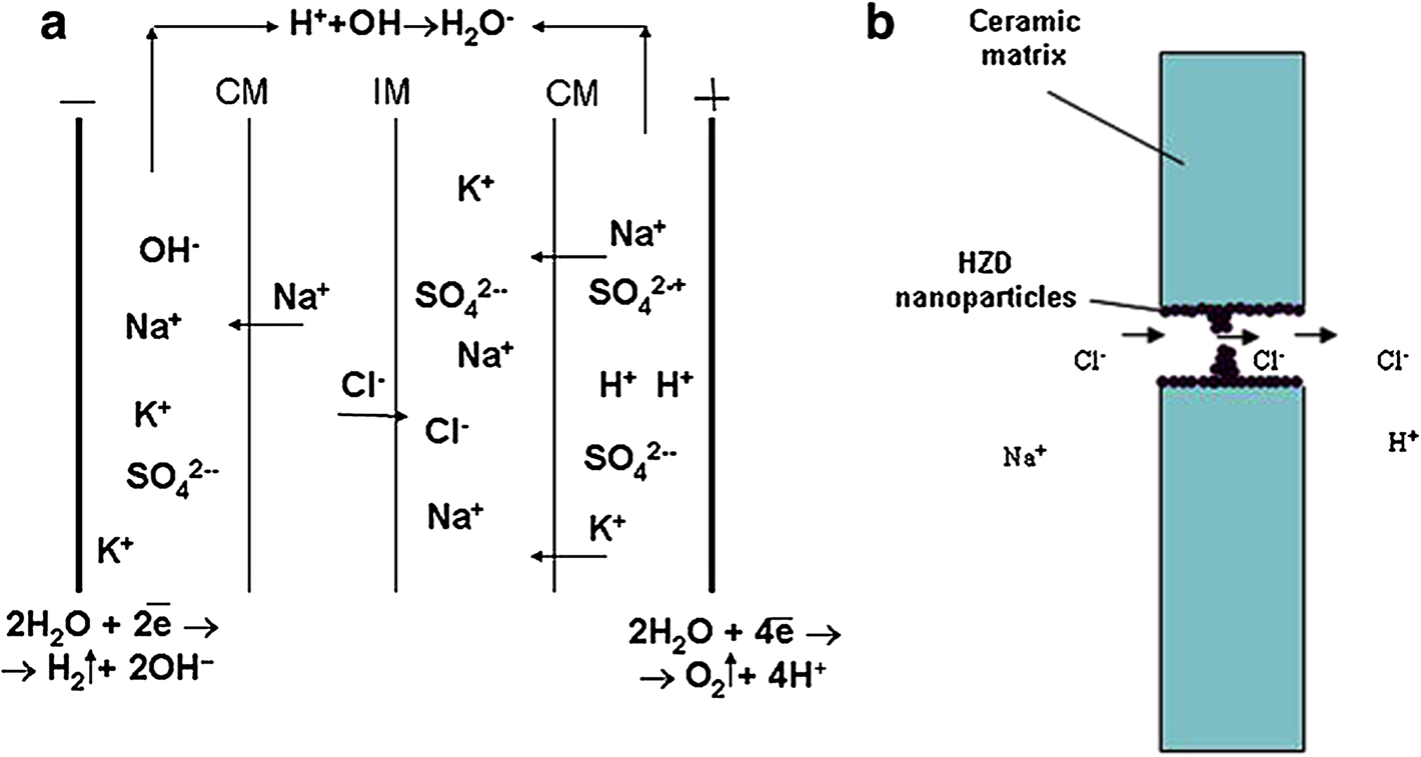
Scheme of ion transport in the membrane system (a) and through the inorganic membrane (b).

Anion exchange function of the inorganic membrane is provided by acidic media from the side of the concentration compartment. Thus, the transport of Na^+^ and Cl^−^ ions was realized through the inorganic and polymer membranes, respectively. Cations and anions accumulated in the concentration compartment. A scheme of ion transport in the membrane system as well as through the inorganic membrane is given in Figure 10.

The limiting current density (*i*_lim_) can be calculated as [25]:

(9) $$i_{\text{lim}}=\mathrm{zF}k_{m}C,$$

where *k*_m_ is the mass transport coefficient, and *z* is the charge number. If the current density (*i*) is higher, than 0.75 *i*_lim_, both species of the solution and ions, which are formed at the membrane-solution interface due to water decomposition (H^+^ and OH^−^), are transported through the membrane. When the centre compartment is filled with glass particles, the following correlation equation can be applied to determine the mass transport coefficient [25]:

(10) $$\mathrm{Sh}=1.52Re^{0.55}Sc^{0.33}$$

where *Sh*, *Re* and *Sc* are the Sherwood, Reynolds and Schmidt criteria, respectively. The criteria can be found as $\mathrm{Sh}=\frac{k\bar{d}}{D}$, Re=ωd¯υ and $\mathrm{Sc}=\frac{\nu }{D}$, where *D* is the diffusion coefficient in a solution (1.6 × 10^−9^ m^2^ s^−1^ for Na^+^ and 2 × 10^−9^ m^2^ s^−1^ for Cl^−^[26]), *v* is the kinematic viscosity (9 × 10^−7^ m^2^ s^−1^[26]), *ω* is the superficial flow velocity (2.5 × 10^−3^ m s^−1^), $\bar{d}$ is the diameter of inert glass particles (6 × 10^−4^ m), the *Re* criterion was estimated as 1.7 and the *Sc* criteria are 562 (Na^+^) and 450 (Cl^−^). Thus, *Sh* ≈ 15 both for cations and anions, and at last, *k*_m_ = 3.7 × 10^−5^ m s^−1^ (Na^+^) and 4.6 × 10^−5^ m s^−1^ (Cl^−^). The process was performed taking into consideration the lower *k*_m_ value, i.e. at 25 A m^−2^, and initial NaCl concentration in the solution (10 mol m^−3^). The results are given in Table 3.

**Table 3:** Electrodialysis of the solution containing NaCl

**Sample**	**After 5 min**		**After 30 min**		**After 60 min**	
	**RD,%**	**CE,%**	**RD,%**	**CE,%**	**RD,%**	**CE,%**
TiO_2_	1	5	7	5	9	3
TiO_2_-HZD-2	17	70	41	28	54	18
TiO_2_-HZD-7	23	95	75	51	95	34

As seen from the table, the current efficiency (CE) decreased in time due to solution depletion. The highest removal degree (RD) and current efficiency were found for the TiO_2_-HZD-7 membrane. This membrane is characterized by the smallest size of pores, which determine charge selectivity. Moreover, the highest surface charge density is reached for this separator.

## Conclusions

The composite inorganic membranes, which contain the active layer of the HZD layer inside coarse-pored ceramics, have been obtained. This has been proved by means of SEM, TEM and SAXS technique. The SCP method followed by resolution of differential pore size distribution, calculations according to homogeneous and heterogeneous geometrical models and potentiometric measurements allow us to determine structure of composite membranes. The approach, which is based on analysis of differential pore size distribution, gives a possibility to recognize each component of a composite. Application of integral pore distribution [12-14] is difficult, when the particle sizes of the constituents are close to each other.

The ceramic matrix is formed mainly with particles of micron size, which are distorted due to annealing and pressure. The ion exchanger consists of nanosized particles, the radius of which is 3 to 5 nm. The nanoparticles form aggregates (*r*_p_ *=* 20 to 23 nm). The larger particles form pores, which are responsible for charge selectivity. Radii of narrowing of these pores have been estimated as 4 to 8 nm; this is in agreement with porosimetry data. Charge selectivity is also due to ion exchange ability of HZD, which is retained under thermal treatment of the membranes. The materials can be used for electromembrane separation; the modified membranes demonstrate higher desalination degree and current efficiency in comparison with the pristine separator. Mechanical stability of the active layer is provided by its location inside pores of ceramics. As expected, the membranes can be used in aggressive media as well as for treatment of solutions containing organic substances.

## Nomenclature

### List of symbols

*A* area (m^2^)

*a* activity (mol m^−3^)

*C* concentration (mol m^−3^)

*D* diffusion coefficient (m^2^ s^−1^)

$\bar{d}$diameter of glass particles (m)

*E*_m_ membrane potential (V)

*F* Faraday constant (96,485 A s mol^−1^)

*I* intensity (cm^−1^)

*i* current density (A m^−2^)

*i*_lim_ limiting current density (A m^−2^)

*k* shape coefficient (dimensionless)

*k*_m_ mass transport coefficient (m s^−1^)

*m* mass of matrix (kg)

*m*^*l*^ mass of modified membrane (kg)

*n* amount of species (mol)

*q* scattering vector (nm^−1^)

*R* gas constant (8.31 J mol^−1^ K^−1^)

*R*_g_ gyration radius (nm)

*r* radius of pores (m, nm)

*r*_*c*_ radius of pore cavities (m, nm)

*r*_*n*_ radius of pore necks (m, nm)

*r*_*p*_ radius of globules (m, nm)

*S* surface (m^2^ kg^−1^)

*S*_m_ surface of a composite membrane (m^2^ kg^−1^)

*T* temperature (K)

*t* transport number through the solution (dimensionless)

*t*_m_ transport number through the membrane (dimensionless)

*V* pore volume (cm^3^ g^−1^)

*V*_micr_ volume of micropores in a matrix (cm^3^ g^−1^)

*V*^/^_micr_ volume of micropores in a matrix (cm^3^ g^−1^)

*z* charge number (dimensionless)

### Greek

*ϵ* porosity of a matrix (dimensionless)

*ϵ*^/^ porosity of a modified membrane (dimensionless)

*ϵ*_d_ dielectric constant (dimensionless);

*ϵ*_p_ porosity due to particles of chosen size (dimensionless)

$\bar{\epsilon }$porosity of ion exchanger (dimensionless)

*ϵ*_0_ dielectric permittivity of free space (8.85 × 10^−12^ F m^−1^)

*η* surface charge density (C m^−2^)

*ν* viscosity (m^2^ s^−1^)

*ρ* electron density (dimensionless)

*ρ*_p_ particle density (kg m^−3^)

*ρ*_b_ bulk density (kg m^−3^)

*τ* time (s)

*ω* linear flow velocity (m s^−1^)

### Dimensionless criteria

*Re* Reynolds number (dimensionless)

*Sc* Schmidt number (dimensionless)

*Sh* Sherwood number (dimensionless)

## Abbreviations

CE: current efficiency (%); HZD: hydrated zirconium dioxide; RD: removal degree (%); SCP: standard contact porosimetry.

## Competing interests

The authors declare that they have no competing interests.

## Authors’ contributions

YSD carried out the mathematical treatment of differential pore size distributions, calculations according to models, experiments dealt to SEM, TEM, potentiometric measurements and electrodialysis. YMV coordinated the study, provided SCP measurements (together with VES and NFN). YPG performed the measurements using the method of small angle X-ray scattering. The manuscript was prepared by YSD and YMV. All authors read and approved the final manuscript.
